# Depression, Anxiety, and Stress among Teachers during the Second COVID-19 Wave

**DOI:** 10.3390/ijerph19105968

**Published:** 2022-05-14

**Authors:** Pablo A. Lizana, Lydia Lera

**Affiliations:** 1 Laboratory of Epidemiology and Morphological Sciences, Instituto de Biología, Pontificia Universidad Católica de Valparaíso, Valparaíso 2373223, Chile; 2 Latin Division, Keiser University, Online Education, Fort Lauderdale, FL 33309, USA; lmarques@keiserunivversity.edu

**Keywords:** COVID-19, depression, anxiety, stress, age, gender, school, teachers

## Abstract

There is a strong background indicating that the teaching profession is one of the most stressful and that their mental health has deteriorated even further during the pandemic. However, there is a little background about the impact of the COVID-19 infection peaks and teachers’ mental health. To this end, 313 teachers were recruited. Via online questionnaires, an evaluation was performed on their depression, anxiety, and stress symptoms on the DASS-21 scale. Teachers’ sociodemographic and socio-personal data were also analyzed. A binary logistic regression was used to analyze the variables which could be associated with each of the symptoms. High rates of depression, anxiety, and stress symptoms were observed among teachers (67%, 73%, and 86%, respectively). Among teachers who were affected by the work–family balance (89%), there was also an increased risk of symptoms of anxiety (OR: 3.2) and stress (OR: 3.5). Depression symptom risk was higher among women (OR: 2.2), and teachers under 35 years old had a risk of presenting all three symptoms (depression OR: 2.2; anxiety OR: 4.0; stress OR 3.0). In contrast, teaching in private educational establishments was a protective factor for anxiety symptoms (OR: 0.3). The results suggest that the second COVID-19 wave profoundly affected teachers’ mental health. Urgent interventions are thus needed to aid teachers’ mental health.

## 1. Introduction

Due to the COVID-19 health crisis, various countries’ governments adopted social distancing and quarantine measures. This has also led to infection peaks varying at different latitudes [1]. Schools were no exception, as they were closed to avoid spreading the disease [2]. In this context, school closures led to a rapid school planning change from in-person to online. This rapid change to online teaching, together with confinement and work–family reconciliation, brought various consequences to teachers’ physical and mental health [3,4]. In this sense, high stress levels have been reported among teachers during the COVID-19 pandemic, along with anxiety, depression, domestic violence, and divorce, all impacting their capacity for proper teaching [5]. Increased anxiety rates among teachers have also been reported, with women presenting higher rates than men [6]. Chinese observers have also reported that stress symptom prevalence among teachers was 9.1%, and that it was important for them to have psychological support [7]. In a study from the beginning of the health crisis in Spain, teachers also reported having work overload, psychosomatic problems, and burnout [8]. Observers have also reported that symptoms between COVID-19 infection peaks saw an increase in stress, anxiety, and depression among the population [9], as well as an emotional response in teachers [10]. Therefore, there is a precedent suggesting that different COVID-19 infection peaks could generate various physical and mental health problems among teachers, but due to the different measures adopted by countries [1] leading to different infection peaks, there could be differences in teacher health impacts which must be reported to generate health policies.

The COVID-19 pandemic context has brought various physical and mental health problems for teachers. Even before the COVID-19 pandemic, it was documented that teaching was one of the worst professions in the world due to health deterioration [11,12,13], with increased mental and physical health deterioration during professional practice, and progressively declining quality of life and psychosocial conditions due to burnout from professional exercise [11,14,15,16]. Critical events such as the COVID-19 pandemic and its various peaks could thus generate important teacher health problems.

Within the Chilean teacher health context, before the pandemic, teachers reported the highest rates of emotional burnout (42.6%) compared to other Latin American countries [17]. Various studies have also indicated the deteriorating quality of life in terms of physical and mental components prior to the COVID-19 health crisis [14,15,18]. However, it has been observed that during the pandemic, Chilean teachers’ mental health component has deteriorated due to teleworking, increased work hours, and low work–family reconciliation, principally among women [4]. A study about engagement and burnout among Chilean teachers compared its results with workers from various occupations and professions before and during the pandemic, revealing that Chilean teachers had less engagement and more professional burnout than other labor groups [18].

Chile saw classes suspended in March 2020, with schools, daycares, and universities closing, along with confinement measures, mobility restrictions, and quarantines [19]. Chile experienced its first major COVID-19 wave roughly around May and June, with important consequences for teachers’ mental and physical health [4,20]. After various months of mobility restrictions and online classes, in 2021, various educational establishments began to hold hybrid-format classes, with face-to-face and online students. However, Chile faced a second wave of COVID-19 during the months from March to June [21], which were the first months of the school year. In this sense, there is little knowledge about the mental health effects on teachers during the second wave of COVID-19. The objective of the following study is thus to analyze the depression, anxiety, and stress levels faced by teachers during the second COVID-19 infection wave, as well as to evaluate the association of sociodemographic and socio-personal factors on the risk of presenting depression, anxiety, and stress symptoms.

We expect to observe high symptomology levels, with younger teachers and female teachers being the most strongly affected. We also anticipate that people with more job instability will present more symptoms.

## 2. Materials and Methods

### 2.1. Participants

This cross-sectional study was performed on a sample of 313 teachers from primary and secondary schools (university professors and technical center teachers were not included in the study) during the COVID-19 pandemic. Measurements were performed during the second COVID-19 wave in Chile, between March and April 2021. Teachers were contacted via email and social media (Facebook and Instagram) following a snowball approach [22]. People who agreed to participate and respond to online surveys gave informed consent via electronic forms. The platform used for the survey was SurveyMonkey (SurveyMonkey, San Mateo, CA, USA). Each participant had to read and sign an electronic informed consent form, where they were invited to voluntary and totally confidential participation, with no remuneration, compensation, or conflict of interest with researchers. The study fulfills all ethical requirements of the Helsinki Declaration and was approved by the Bioethics Committee of Pontificia Universidad Católica de Valparaíso (n°BIOEPUCV-H 393-2021).

The participants then answered questions in two sections: (1) sociodemographic characteristics, such as age, gender, marital status, and city and region of residence within Chile, and work-related information; (2) the survey on stress, anxiety and depression (DASS-21).

### 2.2. Instruments

#### 2.2.1. Work-Related Information

Participating teachers provided information about the funding type of the establishment where they worked (public, private with state subsidies/charter school, or private without state subsidies) and work contract type (fixed-term or indefinite). Regarding the realization of work, they also replied whether they were working 1 = fewer or the same number of hours as before the pandemic (less/equal) or 2 = more (higher), considering the hours where they held classes and all the hours in which teachers prepared materials, revised and created tests, supported students and parents/guardians, and other administrative work typical of the profession.

#### 2.2.2. Personal/Family Life Information

Teachers’ personal information was related to the impact of work in the pandemic on work–family balance. In this question, they had to answer 1 = if family and personal relations were affected due to working, or 2 = family and personal relations were not affected by working.

#### 2.2.3. Depression, Anxiety, and Stress

We employed the Depression, Anxiety, and Stress Scale (DASS-21, [23]), validated in Chile [24]. This instrument includes 21 options with four response options (from 0 = not occurring to 3 = occurring frequently or almost always) grouped into three dimensions: depression (items: 3, 5, 10, 13, 16, 17, and 21), anxiety (items: 2, 4, 7, 9, 15, 19, and 20) and stress (items: 1, 6, 8, 11, 12, 14, and 18). For the study, we used the cutoff points proposed by Lovibond and Lovibond [25]: no symptoms, light symptoms, moderate, severe, and extremely severe [9]. Regarding the reliability of the scale, the Cronbach’s alpha coefficient was α = 0.904 for the depression scale, α = 0.866 for the anxiety scale, and α = 0.874 for the stress scale.

#### 2.2.4. Statistical Analysis

Data were analyzed with STATA 16 software for Windows. Prior to performing the corresponding analysis, data distribution was determined for group difference analysis via the Shapiro–Wilk test. Depression, anxiety, and stress were categorized using the instrument cutoff scores to establish different levels (light, moderate, severe, and extremely severe). First, descriptions were made of both the frequencies and percentages of each category in the DASS-21 dimensions, and the age variable was categorized according to the categories reported by Ozamiz-Etxebarria [26]. Subsequently, a comparison was performed with each of the sociodemographic variables per dimension of the DASS-21 using Student’s t-test, ANOVA, or non-parametric versions (Mann–Whitney test and Kruskal–Wallis test), while for differences between groups, we used the Bonferroni’s post hoc or Dunn tests. To evaluate associations between each DASS-21 dimension and sociodemographic variables, every DASS-21 dimension was divided into two categories (normal and showing a presence of symptoms of the dimension). Finally, a binary logistical regression was performed for each DASS-21 dimension, and to achieve a parsimonious model, we incorporated all variables which gained significance in association analyses by sociodemographic characteristics. Furthermore, confounding gender and age variables were incorporated in all models. The goodness of fit of each logistical regression model was proven with the Hosmer–Lemeshow test.

## 3. Results

The teacher sample comprised 313 teachers, 256 of which were women (81.79%), with an average age of 37.9 (SD 9.92). Table 1 shows that most teachers participating in the study were within the first age range (23–35; 49%), were single (62%), worked in charter schools (40%), and had an indefinite work contract (73%). Regarding work hours during the pandemic, 86% of teachers perceived them to be very high, and 89% were affected by work–family balance.

Table 2 shows that the depression dimension was significantly lower among teachers with spouses/partners and higher among teachers with fixed-term contracts. The anxiety dimension was significantly higher for women (*p* < 0.01), and significantly lower for people with spouses/partners compared to single people and divorced, widow-widowers (DWW). Anxiety was also higher among teachers affected by work–family balance (*p* < 0.01). In the stress dimension, older teachers (>47 years) had a significantly lower score compared with the youngest teachers (*p* < 0.05). Teachers who perceived that they worked more hours during the pandemic and those affected by work–family balance had significantly higher stress scores.

Table 3 presents the distribution of teachers with and without symptoms. Of all teachers, 67% presented depression, 73% presented anxiety, and 86% presented stress. Younger teachers (<35 years) presented the highest rate of depression at 74%. The highest anxiety prevalence was also among young teachers (<35 years) and DWW teachers at 82%. Furthermore, 55% of DWW teachers presented extremely severe anxiety. Younger teachers (<35 years) and single teachers had stress rates of 87% and 90%, respectively. However, there was a notably high rate of DWW teachers with extremely severe stress (41%).

Table 4 presents the distribution of teachers with and without symptoms according to socio-personal variables, where two of the variables are focused on the COVID-19 pandemic situation. Teachers working in public schools presented higher rates of depression, anxiety, and stress than their peers working in charter or private schools (75%, 85% and 90% respectively). In addition, 45% of teachers working in public schools had extremely severe anxiety, compared with 36% among private and charter schoolteachers. In the three studied dimensions, rates were higher among teachers with fixed-term contracts than among teachers with indefinite contracts. However, the highest rate appeared among fixed-contract teachers, where 90% of teachers presented stress. Regarding perceptions on the number of hours worked during the pandemic, the three dimensions showed higher rates for those who felt the hours were greater compared to those who felt that they worked the same amount or less. However, the highest rate was observed in the stress dimension, where 88% of teachers presented stress-associated symptoms. Similarly, regarding work–family reconciliation, affected teachers presented an 88% stress rate.

Table 5 shows the associations between sociodemographic and socio-personal characteristics according to the presence or absence of symptoms. The depression dimension presents significant associations with age, marital status, and contract type. The anxiety and stress dimensions presented significant associations with age, marital status, school type, pandemic hours, and work–family balance.

Table 6 shows the logistic regressions for each of the dimensions for variables which were significant in Table 5. We can observe that people under 35 had the highest risk of presenting depression (OR: 2.15) In the anxiety dimension, for teachers affected by work–family balance, those under 35 and females had the highest risk. However, one observable protective factor against presenting anxiety was working at a private school (OR: 0.29). In contrast, teachers working in regular public municipal schools presented a higher risk of anxiety (OR: 3.48, 95% CI: 1.62–7.44 *p* < 0.001, data not shown). Finally, teachers under 35 and those affected by work–family balance had the highest stress risk.

## 4. Discussion

This study presents a high rate of teachers suffering from the symptoms of depression, anxiety, and stress (67%, 73%, and 86%, respectively), which was higher among women and teachers under 35. One relevant result is the high rate of women reported in this study (82%). Teaching is a highly feminized profession, with a few exceptions [27], and Chile follows this pattern [14,28]. These results are relevant in the perspective of interventions among teachers, since it has already been observed that during the pandemic, females were principally affected in terms of their mental health [20].

We can observe that teachers’ depression, anxiety, and stress levels were rather high (67%, 73%, and 86%, respectively) compared to prior studies [9,29,30]. One study on teachers, performed 6 months after schools were closed, reported a 32.2% rate of some degree of depressive symptoms, principally among women and in the ‘Light’ category. The present study showed symptoms almost twice as high as what Ozamiz-Etxebarria et al. presented, and the depressive symptom category was higher into the moderate and extremely severe areas [26]. We observed significant differences between teachers with stable contracts and those without such contracts, which led to a significant association with depressive symptoms (see Table 5). In alignment with these results, studies from the COVID-19 pandemic have indicated that work instability negatively impacts teachers’ mental health [26], as well as young adults, due to financial uncertainty [29]. Similar results were also observed before the pandemic, where teachers with unstable contracts showed worse mental health [30]. This sharp rise in depressive symptoms may be due to the period when the teachers were evaluated, corresponding to the beginning of the school year after experiencing the first year of the pandemic, where they were facing new changes. This phenomenon of change and a lack of control has been associated with depressive symptoms [31].

A recent review indicated high depression, anxiety, and stress rates among teachers (19%, 17%, and 30%, respectively) [32]. However, our results reported higher rates. These worrying results may be attributed to the fact that Chilean teachers carry higher stress loads compared to those in Argentina, Peru, Mexico, and Uruguay [17]. Chilean teachers also present the highest rates of high emotional burnout compared to Argentina, Uruguay, Peru, Mexico, and Ecuador [16,17]. Similarly, comparative results from before and during the COVID-19 pandemic among Chilean teachers reported that pre-pandemic teachers suffered from deteriorated mental health, with mental health declining significantly during the pandemic [18,20]. These results could indicate that Chilean teachers suffered from deteriorated mental health before the pandemic, and during the pandemic, their mental health became even worse. Regarding the second wave of the pandemic, it has been observed that emotional reactions and poor mental health rose from 27 to 84% in an adjusted model among teachers [10]. During events such as school reopening, peak anxiety points have also been observed among teachers [33]. Another relevant variable for anxiety symptoms is gender. The following study observed that anxiety risk increased by 2.08 times among women compared with men, coinciding with previous research where women had higher anxiety rates than their male counterparts [6,29,34]. These results are relevant since most teachers are women [28]. It has also been observed that women are more likely to suffer over work–family balance than their male peers, although they share the same esteem for performing both family and professional roles [35]. In the present study, the results showed that working in private schools was a protective factor against anxiety symptoms for teachers (OR: 0.29). These results align with pre-pandemic reports where private school teachers had fewer psychological demands, lower the risk of presenting a less active job and increasing possibilities of professional development compared to public municipal schools [36]. In contrast, public schoolteachers had a higher anxiety risk (OR: 3.48), which could be related with the high workloads teachers perceived compared with charter and private schoolteachers [34]. According to our data, it is possible that pre-pandemic occupational health differences may have increased, impacting public schoolteachers’ mental health.

Regarding age, we observed that most teachers in our sample were under 47 (68.7%), which agrees with data from the Chilean Education Ministry where 61.4% of teachers were under 44 years old [28]. Therefore, if young adult teachers present health problems, they constitute a large percentage of the total teacher population. In this line, we observed that teachers under 35 had an increased risk of suffering from the three symptoms (depression, anxiety, stress). These worrying results align with studies during the pandemic, which observed that young adults have experienced mental health impacts from various measures generated by countries to contain COVID-19 infections. A study in 63 countries found that young adults are face the highest risk of suffering the symptoms of depression, anxiety, and stress (Varma et al., 2021) High rates of generalized anxiety disorder have been reported compared with older people [35]. The Australian population also showed that younger people showed higher rates of negative emotions [37]. This phenomenon observed in the general population has also been described among teachers, where younger teachers had higher stress levels compared to their more mature colleagues [26]. These results may be attributed to various previously reported factors, such as the high amount of information which young people have within their reach, which, when consumed for long periods, can include false information and generate mental health problems [35]. It has also been observed that among young adults, loneliness and economic problems are associated with worse outcomes in depression and anxiety, respectively [29]. These data align with our findings where people with fixed-term contracts have higher depression risks (OR: 1.4). In contrast, some protective factors have been observed which help decrease depressive symptoms among young adults, such as increased physical activity [29]. However, pre-pandemic teachers already reported a lack of time to perform physical activity due to the work they had to do at home [38]. During the pandemic, 86% of teachers said that their working hours increased, impeding any activity to promote teacher wellbeing.

High stress and anxiety levels could also be due to classroom educational technology use [39,40]. During the first wave in Chile, it was observed that teleworking was a factor affecting teachers’ quality of life [4]. In the case of the second COVID-19 wave when this study was performed, hybrid classes had been implemented. To this end, reports from Spain from several months prior stated that the implementation of face-to-face classes generated high depression, anxiety, and stress levels among teachers [26]. It is therefore relevant for policy decision-makers to include existing evidence about teachers’ physical and mental health. These mental health problems due to technology use may arise due to a lack of training in educational technologies, along with pressure to use this technology in professional practice [40]. In the case of the second wave in Chile, teachers had to deal with hybrid classes (part of the students at home and part in class), where, once again, they were pressured by working with students in both locations simultaneously.

Finally, we suggest generating policies and interventions which help maintain decent mental health for teachers and reverse the impacts produced by the COVID-19 health crisis. Even before the pandemic, it was observed that teaching was a highly stressful profession, and during the first wave, teachers’ quality of life decreased significantly (Lizana et al., 2021). Data from various sources indicate that stress, anxiety, and depression levels during the health crisis are drastically higher than those typically observed among teachers [32]. Therefore, it is necessary to protect teachers’ mental and physical health given that their behavior may predict students’ emotional wellbeing and commitment.

### Limitations

This study has some limitations which must be described to properly interpret its results. First, the nature of the study must be considered given that it is cross-sectional and only provides a snapshot of the participants. This can influence the response at the moment they reply, which means that no cause–effect relations can be made. Second, we used snowball sampling; non-probabilistic sampling has inherent limitations for representing an entire population. Finally, we do not have data on the medical history of the respondents, an aspect that may also influence the results. Some strengths of the study are that it is the first about depression, anxiety, and stress levels among schoolteachers during the second COVID-19 wave in Chile.

## 5. Conclusions

High depression, anxiety, and stress levels were observed in the studied teacher sample, and were highest among women and people under 35. These results suggest teachers’ mental health deterioration during the pandemic. This study reported some negative impacts of the second COVID-19 pandemic wave on teachers’ mental health, and the factors increasing the risk of symptoms with negative effects on work–family balance. The present results are a useful resource for future interventions among teachers to help improve their mental health.

## Figures and Tables

**Table 1:** Sociodemographic characteristics of the sample.

Variable	Total	Male	Female	*p*
**Age (years)**				
23–35	152 (48.56)	29 (50.88)	123 (48.05)	0.857 ^a^
36–46	98 (31.31)	18 (31.58)	80 (31.25)	
47 years or older	63 (20.13)	10 (17.54)	53 (20.70)	
**Marital status**				
Single	193 (61.66)	36 (63.16)	157 (61.33)	0.706 ^b^
Married/partnered	98 (31.31)	16 (28.07)	82 (32.03)	
DWW	22 (7.03)	5 (8.77)	17 (6.64)	
**Type of school**				
Public (state)	91 (29.07)	20 (35.09)	71 (27.73)	0.520 ^a^
Private (subsidized)	125 (39.94)	20 (35.09)	105 (41.02)	
Private (nonsubsidized)	97 (30.99)	17 (29.82)	80 (31.25)	
**Type of contract**				
Fixed	86 (27.48)	17 (29.82)	69 (26.95)	0.661 ^a^
Indefinite	277 (72.52)	40 (70.18)	187 (73.05)	
**Work hours in pandemic ^c^**				
Less/Equal	43 (13.74)	10 (17.54)	33 (12.89)	0.356 ^a^
Higher	270 (86.26)	47 (82.46)	223 (87.11)	
**Work-family balance**				
Unaffected	34 (10.86)	10 (17.54)	24 (9.38)	0.073 ^a^
Affected	279 (89.14)	47 (82.46)	232 (90.63)	

DWW, Divorced Widow Widower; ^a^ Chi-squared; ^b^ Fisher’s exact test; c Work hours in the pandemic, Higher: more than before the pandemic, Less/Equal: less than or equal before the pandemic.

**Table 2:** Sociodemographic characteristics according to the dimensions of DASS-21.

Variable	Depression	*p*	Anxiety	*p*	Stress	*p*
**Gender**						
Male (n 57)	7.67 ± 5.63	0.543	6.18 ± 4.89	**0.008**	12.07 ± 4.61	0.090
Female (n 256)	8.12 ± 5.61		8.25 ± 5.33		13.10 ± 4.84	
**Age (years)**						
23–35 (n 152) (a)	8.45 ± 5.38	0.257	8.44 ± 4.93	0.082	13.23 ± 4.44	**0.037**
36–46 (n 98) (b)	7.81 ± 5.46		7.39 ± 5.06		13.44 ± 4.61	*Post hoc*
47 years or older (n 63) (c)	7.38 ± 6.34		7.38 ± 6.34		11.33 ± 5.65	c < a, b
**Marital status**						
Single (n 193) (a)	8.54 ± 5.55	**0.024**	8.30 ± 5.21	**0.043**	13.28 ± 4.49	0.068
Married/partnered (n 98) (b)	6.86 ± 5.67	*Post hoc*	6.85 ± 5.46	*Post hoc*	11.98 ± 5.07	
DWW (c)	8.91 ± 5.30	a > b; c > b	8.68 ± 5.02	a > b. c > b	11.77 ± 5.90	
**Type of school**						
Public (state. n 91) (a)	8.49 ± 5.47	0.306	9.00 ± 5.40	**0.018**	13.40 ± 4.17	0.362
Private (subsidized. n 125) (b)	8.26 ± 5.87		7.86 ± 5.05	*Post hoc*	13.09 ± 4.75	
Private (nonsubsidized. n 97) (c)	7.31 ± 5.37		6.83 ± 5.38	a > c	12.24 ± 5.39	
**Type of contract**						
Fixed (n 86)	9.35 ± 5.94	**0.015**	7.70 ± 5.25	0.359	13.55 ± 4.75	0.116
Indefinite (n 277)	7.54 ± 5.41		8.34 ± 5.45		12.67 ± 4.82	
**Work hours in pandemic**						
Less/Equal (n 43)	7.74 ± 5.71	0.644	6.70 ± 5.82	0.071	10.47 ± 5.06	**0.001**
Higher (n 270)	8.08 ± 5.60		8.06 ± 5.21		13.30 ± 5.21	
**Work–family balance**						
Unaffected (n 34)	7.35 ± 6.06	0.338	5.74 ± 5.32	**0.008**	9.94 ± 4.66	**<0.001**
Affected (n 279)	8.12 ± 5.56		8.13 ± 5.25		13.28 ± 4.71	

DWW, Divorced Widow Widower. Mann–Whitney test, K–Wallis test with post hoc comparison Dunn’s test.

**Table 3:** Distribution of teachers with different symptomatology (mild, moderate, severe, and extremely severe) according to gender, age, and marital status.

	Total	Gender		Age (Years)			Marital Status		
		Male	Female	<35	36–46	>47 Years	Single	Married	DWW
**Depression**									
Normal	104 (33.23)	19 (33.33)	85 (33.20)	40 (26.32)	35 (35.71)	29 (46.03)	56 (29.02)	43 (43.88)	5 (22.73)
Mild	35 (11.18)	7 (12.28)	28 (10.94)	20 (13.16)	10 (10.20)	5 (7.94)	19 (9.84)	13 (13.27)	3 (13.64)
Moderated	71 (22.68)	16 (28.07)	55 (21.48)	43 (28.29)	21 (21.43)	7 (11.11)	53 (27.46)	14 (14.29)	4 (18.18)
Severe	40 (12.78)	6 (10.53)	34 (13.28)	17 (11.18)	14 (14.29)	9 (14.29)	26 (13.47)	9 (9.18)	5 (22.73)
Extremely severe	63 (20.13)	9 (15.79)	54 (21.09)	32 (21.05)	18 (18.37)	13 (20.63)	39 (20.21)	19 (19.39)	5 (22.73)
**Anxiety**									
Normal	83 (26.52)	21 (36.84)	62 (24.22)	27 (17.76)	29 (29.5)	27 (42.86)	41 (21.24)	38 (38.78)	4 (18.18)
Mild	21 (6.71)	5 (8.77)	16 (6.25)	10 (6.58)	8 (8.16)	3 (4.76)	12 (6.22)	7 (7.14)	2 (9.09)
Moderated	55 (17.57)	11 (19.30)	44 (17.19)	31 (20.39)	18 (18.37)	6 (9.52)	38 (19.69)	14 (14.29)	3 (13.64)
Severe	33 (10.54)	3 (5.26)	30 (11.72)	22 (14.47)	8 (8.16)	3 (4.76)	25 (12.95)	7 (7.14)	1 (4.55)
Extremely severe	121 (38.66)	17 (29.82)	104 (40.63)	62 (40.79)	35 (35.71)	24 (38.10)	77 (39.90)	32 (32.65)	12 (54.55)
**Stress**									
Normal	43 (13.74)	8 (14.04)	35 (13.67)	14 (9.21)	13 (13.27)	16 (25.40)	19 (9.84)	20 (20.41)	4 (18.18)
Mild	31 (9.90)	9 (15.79)	22 (8.59)	13 (8.55)	6 (6.12)	12 (19.05)	17 (8.81)	14 (14.29)	0 (0)
Moderated	62 (19.81)	14 (24.56)	48 (18.75)	37 (24.34)	17 (17.35)	8 (12.70)	46 (23.83)	13 (13.27)	3 (13.64)
Severe	95 (30.35)	16 (28.07)	79 (30.86)	49 (32.24)	35 (35.71)	11 (17.46)	59 (30.57)	30 (30.61)	6 (27.27)
Extremely severe	82 (26.20)	10 (17.54)	72 (28.13)	39 (25.66)	27 (27.55)	16 (25.40)	52 (26.94)	21 (21.43)	9 (40.91)

DWW, Divorced Widow Widower.

**Table 4:** Distribution of teachers with different symptomatology (mild, moderate, severe, and extremely severe) according to socio-personal variables.

	Type of School			Type of Contract		Work Hours in Pandemic		Work-Family Balance	
	Public	Private S ^a^	Private NS ^b^	Fixed	Indifinite	Less/Equal	Higher	Unaffected	Affected
**Depression**									
Normal	23 (25.27)	46 (36.80)	35 (36.08)	21 (24.42)	83 (36.56)	12 (35.29)	92 (32.97)	12 (27.91)	92 (34.07)
Mild	13 (14.29)	9 (7.20)	13(13.4)	11 (12.79)	24 (10.57)	6 (17.65)	29 (10.39)	7 (16.28)	28 (10.37)
Moderated	23 (25.27)	27 (21.60)	21 (21.65)	16 (17.60)	55 (24.23)	7 (20.59)	64 (22.94)	13 (30.23)	58 (21.48)
Severe	13 (14.29)	13 (10.40)	14 (14.43)	12 (13.95)	28 (12.33)	1 (2.94)	39 (13.98)	2 (4.65)	38 (14.07)
Extremely severe	19 (20.88)	30 (24.00)	14 (14.43)	26 (30.23)	37 (16.3)	8 (23.53)	55 (19.71)	9 (20.93)	54 (20.00)
**Anxiety**									
Normal	14 (15.38)	32 (25.6)	37 (38.14)	20 (23.26)	63 (27.75)	17 (50.0)	66 (23.66)	17 (39.53)	66 (24.44)
Mild	9 (9.89)	7 (5.6)	5 (5.15)	6 (6.98)	15 (6.61)	1 (2.94)	20 (7.17)	2 (4.65)	19 (7.04)
Moderated	18 (19.78)	26 (20.8)	11 (11.34)	16 (18.60)	39 (17.18)	5 (14.71)	50 (17.92)	8 (18.60)	47 (17.41)
Severe	9 (9.89)	15 (12)	9 (9.28)	10 (11.63)	23 (10.13)	3 (8.82)	30 (10.75)	4 (9.30)	29 (10.74)
Extremely severe	41 (45.05)	45 (36)	35 (36.08)	34 (39.53)	87 (38.33)	8 (23.53)	113 (40.36)	12 (27.91)	109 (40.37)
**Stress**									
Normal	9 (9.89)	13 (10.4)	21 (21.65)	9 (10.34)	34 (14.98)	11 (32.35)	32 (11.47)	11 (25.58)	32 (11.85)
Mild	4 (4.40)	16(12.8)	11 (11.34)	6 (6.98)	25 (11.01)	4 (11.76)	27 (9.68)	5 (11.63)	26 (9.63)
Moderated	18 (19.78)	28 (22.4)	16 (16.49)	16 (18.60)	46 (20.26)	8 (23.53)	54 (19.35)	10 (23.26)	52 (19.26)
Severe	38 (41.76)	34 (27.2)	23 (23.71)	30 (34.88)	65 (28.63)	9 (26.47)	86 (30.82)	12 (27.91)	83 (30.74)
Extremely severe	22 (24.18)	34 (27.2)	26 (26.8)	25 (29.07)	57 (25.11)	2 (5.88)	80 (28.67)	5 (11.63)	77 (28.52)

^a^ Private with state subsidies/charter school; ^b^ Private without state subsidies.

**Table 5:** Sociodemographic and socio-personal features of Chilean teachers according to the presence or absence of symptomatology.

Variable	Normal	Depression	Normal	Anxiety	Normal	Stress
**Gender**						
Male	19 (18.27)	38 (18.18)	21 (25.30)	36 (15.65)	8 (14.04)	49 (18.15)
Female	85 (81.73)	171 (81.82)	62 (74.70)	194 (84.35)	35 (81.40)	221 (81.85)
**Age (years)**						
23–35	40 (38.46)	112 (53.59) *	27 (32.53)	125 (54.35) ***	14 (32.56)	138 (51.11) **
36–46	35 (33.65)	63 (30.14)	29 (34.94)	69 (30.00)	13 (30.23)	85 (31.48)
47 years or older	29 (27.88)	34 (16.27)	27 (32.53)	36 (15.65)	16 (37.21)	47 (17.41)
**Marital status**						
Single	56 (53.85)	137 (65.55) *	41 (49.40)	152 (66.09) **	19 (44.19)	174 (64.44) *
Married/partnered	43 (41.35)	55 (26.32)	38 (45.78)	60 (26.09)	20 (46.51)	78 (28.89)
DWW	5 (4.81)	17 (8.13)	4 (4.82)	18 (7.83)	4 (9.30)	18 (6.67)
**Type of school**						
Public (state)	23 (22.12)	68 (32.54)	14 (16.87)	77 (33.48) **	9 (20.93)	82 (30.37) *
Private (subsidized)	46 (44.23)	79 (37.80)	32 (38.55)	93 (40.43)	13 (30.23)	112 (41.48)
Private (nonsubsidized)	35 (33.65)	62 (29.67)	37 (44.58)	60 (26.09)	21 (48.84)	76 (28.15)
**Type of contract**						
Fixed	83 (79.81)	144 (68.90) *	63 (75.90)	164 (71.30)	34 (79.07)	193 (71.48)
Indefinite	21 (20.19)	65 (31.10)	20 (24.10)	66 (28.70)	9 (20.93)	77 (28.52)
**Work hours in pandemic ^a^**						
Less/Equal	12 (11.54)	31 (14.83)	17 (20.48)	26 (11.30) *	11 (25.58)	32 (11.85) **
Higher	92 (88.46)	178 (85.17)	66 (79.52)	204 (88.70)	32 (74.42)	238 (88.15)
**Work–family balance**						
Unaffected	12 (11.54)	22 (10.53)	17 (20.48)	17 (7.39) ***	11 (25.58)	23 (8.52) ***
Affected	92 (88.46)	187 (89.47)	66 (79.52)	213 (92.61)	32 (74.42)	247 (91.48)

DWW, Divorced Widow Widower; ^a^ Higher: more than before the pandemic, Less/Equal: less than or equal before the pandemic. * *p* < 0.05, ** *p* < 0.01, *** *p* < 0.001, Chi-squared test.

**Table 6:** Logistical regressions of the presence/absence of symptomatology with sociodemographic and socio-personal variables adjusted by gender and age.

	Depression		Anxiety		Stress	
	OR [95% CI] ^a^	*p*	OR [95% CI] ^a^	*p*	OR [95% CI] ^a^	*p*
**Marital status**						
Single (n 193) (a)	1		1		1	
Married/partnered (n 98) (b)	0.68 [0.40–1.19]	0.181	0.63 [0.35–1.17]	0.145	0.58 [0.27–1.26]	0.173
DWW (c)	2.10 [0.70–6.31]	0.188	2.84 [0.78–10.38]	0.133	0.93 [0.24–3.60]	0.914
**Type of school**						
Public (state)	-	-	1		1	
Private (subsidized)	-	-	0.47 [0.22–1.01]	0.052	0.89 [0.35–2.30]	0.817
Private (nonsubsidized)	-	-	0.29 [0.13–0.62]	**0.001**	0.43 [0.18–1.05]	0.064
**Type of contract** (Fixed)	1.40 [0.77–2.54]	0.268	-	-	-	-
**Work hours in pandemic**	-	-	1.66 [0.82–3.49]	0.223	1.81 [0.71–4.59]	0.212
**Work–family balance**	-	-	3.16 [1.31–7.66]	**0.011**	3.48 [1.30–9.31]	**0.013**
**Age (years)**						
47 years or older	1		1		1	
35–46 years	1.55 [0.80–3.03]	0.191	1.69 [0.82–3.49]	0.153	1.73 [0.73–4.11]	0.212
<35	2.15 [1.07–4.28]	**0.030**	3.98 [1.84–8.59]	**<0.001**	2.96 [1.17–7.47]	**0.022**
**Gender** (female)	1.08 [0.58–2.01]	0.812	2.08 [1.06–4.09]	**0.033**	0.97 [0.40–2.37]	0.951
Hosmer-Lemeshow test ^b^	0.495		0.490		0.319	

^a^ OR. Odds Ratios [Confidence interval]; ^b^ A value above 0.05 indicates that the model fits the data; DWW, Divorced Widow Widower.

## Data Availability

The datasets generated and/or analyzed during the current study are available from the corresponding author on reasonable request.
